# Molecular Mechanisms of p53 Deregulation in Cancer: An Overview in Multiple Myeloma

**DOI:** 10.3390/ijms17122003

**Published:** 2016-11-30

**Authors:** Ana B. Herrero, Elizabeta A. Rojas, Irena Misiewicz-Krzeminska, Patryk Krzeminski, Norma C. Gutiérrez

**Affiliations:** 1 Cancer Research Center-IBMCC (USAL-CSIC), 37007 Salamanca, Spain; anah@usal.es (A.B.H.); elirr@usal.es (E.A.R.); irenamk@usal.es (I.M.-K.); patrykk@usal.es (P.K.); 2 Institute of Biomedical Research of Salamanca (IBSAL), 37007 Salamanca, Spain; 3 National Medicines Institute, 00725 Warsaw, Poland; 4 Hematology Department, University Hospital of Salamanca, 37007 Salamanca, Spain

**Keywords:** p53, myeloma, cancer, *TP53* mutations, epigenetics, *TP53* methylation, *TP53* splicing, miRNAs, p53-based therapy

## Abstract

The p53 pathway is inactivated in the majority of human cancers. Although this perturbation frequently occurs through the mutation or deletion of p53 itself, there are other mechanisms that can attenuate the pathway and contribute to tumorigenesis. For example, overexpression of important p53 negative regulators, such as murine double minute 2 (MDM2) or murine double minute 4 (MDM4), epigenetic deregulation, or even alterations in *TP53* mRNA splicing. In this work, we will review the different mechanisms of p53 pathway inhibition in cancer with special focus on multiple myeloma (MM), the second most common hematological malignancy, with low incidence of p53 mutations/deletions but growing evidence of indirect p53 pathway deregulation. Translational implications for MM and cancer prognosis and treatment are also reviewed.

## 1. Introduction

Since its discovery in 1979, the tumor suppressor protein p53 has been extensively studied. p53 is a transcription factor activated in response to several forms of cellular stress including DNA damage, hypoxia, viral infection, heat shock, and mitogenic or oncogenic stresses among others [1,2,3]. p53 pathway activation is mediated by the protein stabilization, via the disruption of a p53/murine double minute 2 (MDM2) negative feedback loop, and is further enhanced by many types of post-translational modifications, such as phosphorylation, acetylation, sumoylation, ubiquitination, and methylation [4]. Once activated, p53 induces the expression of many target genes that mediate several tumor suppressor mechanisms, such as the cell cycle arrest (p21, Gadd45, and 14-3-3σ), programmed cell death (Bax, PUMA, and Noxa), senescence (p21), and inhibition of angiogenesis (TSP1 and maspin) [5,6]. Recently, non-canonical activities of p53 that affect cell metabolism, autophagy and necrosis have started to emerge, and several evidences suggest that tumor suppressive effects of p53 extend beyond its ability to arrest cell cycle or induce programmed cell death [7].

Nevertheless, the importance of p53 in tumor suppression is unequivocal, as shown by its mutation in more than half of all sporadic human cancers, the susceptibility to cancer of individuals with Li–Fraumeni syndrome who inherit a mutant *TP53* allele, and the spontaneous cancer predisposition of *Trp53*-null mice [8,9].

Interestingly, there are some tumors that inactivate the pathway almost exclusively through direct p53 mutation or deletion of its locus, like esophageal squamous carcinoma [10], whereas others are known to abrogate the p53 pathway by other type of mechanisms, such as the amplification of important negative p53 regulators, that have been reported in the majority of liposarcomas, melanomas and retinoblastomas [11]. In hematological malignancies, p53 mutations and deletions are uncommon events [12,13,14,15,16]. However, p53 may play a broader role in the pathogenesis or progression of these diseases than previously thought, since an imbalance in p53 activity can also be generated through post-transcriptional or post-translational mechanisms.

**Figure 1:**
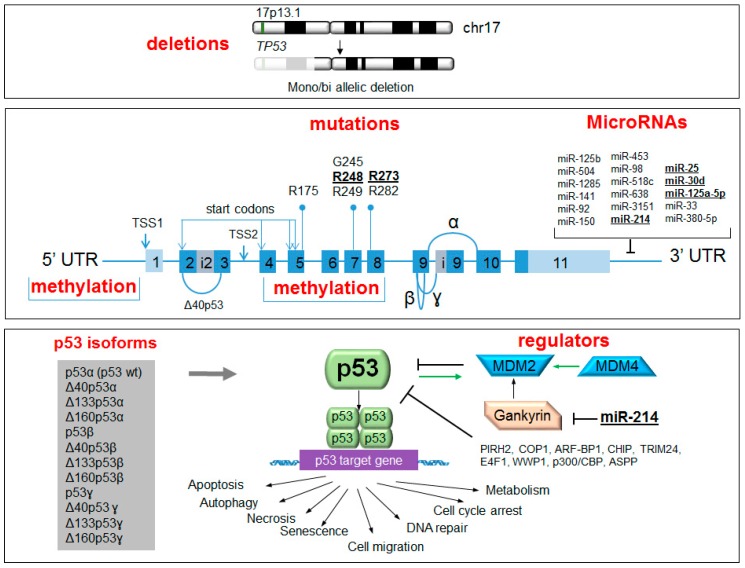
Mechanisms of p53 regulation in cancer. p53 can be attenuated directly, by mutation or deletion, or indirectly through alterations in methylation, miRNAs, isoform expression and p53 regulators. Six *TP53* hotspot mutations and regions potentially affected by methylation are indicated. p53 isoforms arise from the use of two alternative transcription start sites (TSS1 and TSS2), four start codons and alternative splicing, which originates the isoforms α, β and γ. miRNAs and regulators reported to affect p53 expression are shown. Alterations reported in MM are highlighted in bold and underlined.

This review summarizes the different molecular alterations that can contribute to the attenuation of p53 pathway in cancers with special focus on multiple myeloma (MM) (Figure 1). This cancer is the second most frequent hematological malignancy, with an approximate incidence of 4–5 cases per 100,000 inhabitants per year [17]. MM is characterized by the accumulation of immunoglobulin-secreting clonal malignant plasma cells within the bone marrow, leading to cytopenias, bone resorption and renal damage. It is a clinically and biologically heterogeneous disease with a broad spectrum of genetic abnormalities that mainly include translocations, copy number abnormalities and mutations [18]. Although MM initially responds to chemotherapy, continuous recurrences are part of its natural history, so the search for new drugs that control the disease is required [19]. Accordingly, the potential use of p53 reactivating agents in the treatment of MM and other cancer diseases will be highlighted.

## 2. Alterations of *TP53* Gene in Human Cancers and Particularly in Multiple Myeloma (MM)

The tumor suppressor p53 is mutated in approximately 50% of human cancers [6,20]. *TP53* mutations are distributed mainly in coding exons with a strong predominance for exons 4–9, which encode the DNA-binding domain of the protein. In fact, 95% of the *TP53* mutations stand in the core DNA-binding domain [21]. *TP53* mutations can be classified in two categories: contact and structural mutations [22]. Contact mutations affects residues involved directly in DNA-contacts without altering p53 folding, and perturb the transcriptional function of p53 protein. In contrast, structural mutations lead to destabilization of the local structure of p53 core domain [23,24,25]. In all types of human cancers, the missense *TP53* mutations have been detected predominantly in 6 hotspot residues located within the DNA-binding domain (residues R175, G245, R248, R249, R273, and R282) [26,27].

Although the frequency of *TP53* mutations in hematological malignancies is generally lower than in solid neoplasms, they have been reported in Burkitt‘s lymphomas, chronic myeloid leukemia, adult T-cell leukemia, B-cell prolymphocytic leukemia and chronic lymphocytic leukemia (CLL) [12,13,14,15]. In MM, *TP53* mutations are uncommon at diagnosis (~8%) [28,29,30], although the incidence increases in the advanced stages of the disease, suggesting its essential role in MM progression [31,32]. These findings have also been confirmed by using massively parallel sequencing [16,33]. Thus, only 8% of MM patients analyzed by Lohr et al. showed missense *TP53* mutations, two of which corresponded to hotspot residues R273 and R248 within the DNA-binding domain, and commonly mutated in other human tumors [33]. In contrast, the frequency of *TP53* mutations increases up to 25% in plasma cell leukemia [16]. Furthermore, longitudinal analysis of MM patients reveals that *TP53* mutations are often acquired at relapse [16].

On the other hand, deletion of the chromosomal region 17p13, containing the *TP53* gene locus, is a recurrent cytogenetic abnormality in MM present in around 10% of new cases. It is one of the most powerful prognostic factors associated with unfavorable outcome [34,35,36,37,38,39]. Moreover, *TP53* deletion has been associated with resistance to chemotherapy and early evolution to extramedullary disease [39,40,41,42,43,44]. *TP53* deletions remain a negative prognostic factor marker after high-dose chemotherapy plus autologous stem cell transplantation [45], and even when bortezomib-based induction has been used [46]. The relevance of the clonal size carrying del(17p) is currently a matter of debate. Several studies suggest that del(17p) is a survival predictor only if the abnormality is present in more than 50%–60% of the cells [43,47,48]. Interestingly, Lodé et al. reported that *TP53* mutations were exclusively associated with del(17p) [28]. Furthermore, it has been recently reported that biallelic inactivation of *TP53* is more common in aggressive plasma cell neoplasms, and is frequently seen at relapse leading to rapid progression to plasma cell leukemia and/or extramedullary disease [16,49]. All these findings highlight the critical value of *TP53* deletion and/or mutation in the pathogenesis of MM and reinforce the necessity of new therapeutic approaches for these high-risk patients.

## 3. Alterations of p53 Regulators

The most known mechanism to indirectly attenuate or disable p53 activity is through overexpression of their negative regulators. The best studied is MDM2 (murine double minute 2), which was originally identified in a double minute chromosome present in the murine tumorigenic cell line 3T3-DM [50]. MDM2 controls p53 through two distinct mechanisms: by directly binding to the p53 N-terminal transactivation domain, which prevents its transcriptional activity, and by promoting p53 proteasomal degradation [51,52]. MDM2 contains a RING finger domain that after binding to an E2 ubiquitin-conjugating enzyme, promote the ubiquitination of p53, which marks the protein for proteasomal degradation. The interaction of MDM2 with p53 also mediates the translocation of p53 from the nucleus to the cytoplasm, which favors its rapid degradation by the proteasome. Under normal physiological conditions, p53 has a very short half-life, due to the continuous degradation by MDM2. After different stress signals, the MDM2–p53 interaction is uncoupled by multiple mechanisms, such as phosphorylation, acetylation, redistribution of protein complexes and modifications in the subcellular localization, which result in p53 pathway activation [51,52,53].

The essential role of MDM2 in the negative regulation of p53 was clearly illustrated in mouse models [54]. In agreement with the results obtained in mice, overexpression of *MDM2* seems to be a common mechanism by which human cancers abrogate the p53 pathway. *MDM2* upregulation has been found in various types of cancer [55], including MM [56]. In a study carried out in a series of 82 MM patients, *MDM2* amplification, resulting from chromosome 12 diploidy, was seen in 8% of the cases, while another 8% had trisomy 12 with an equivalent increase in signals for *MDM2* [40]. Overexpression of *MDM2* has been found in myeloma cell lines and primary samples obtained from patients with MM and plasma cell leukemia. Moreover, inhibition of MDM2 was found to trigger MM cell apoptosis [57].

Genetic amplification and inheritance of *MDM2* promoter single-nucleotide polymorphisms (SNPs) are the two best-studied mechanisms for up-regulating MDM2 activity [58]. However, this activity may also be influenced by alterations in proteins that regulate MDM2 and also by some microRNAs [59]. Examples of MDM2 regulators are: ARF4 that may dampen the p53 pathway by releasing MDM2; HAUSP that targets MDM2 resulting in its stabilization and negative regulation of the p53 pathway; and WIP1, a phosphatase that dephosphorylates MDM2 preventing prolonged p53-pathway activation [11]. Amplifications of these factors have been detected at different percentages in some tumors by The Cancer Genome Atlas Network (TCGA) and also by several research groups [11]. To our knowledge, there are no published data about alterations of MDM2 protein regulators in MM.

MDM4 is also an important negative regulator of the p53 pathway, which was identified in a screen for p53-interacting proteins in 1996 by Shvarts et al. [60]. MDM4 seems to inhibit p53 transcriptional activity by two ways: through direct binding of its N-terminal domain to p53 [61], and also by forming heterodimers with MDM2 through RING-RING domain interactions. These interactions have been reported to enhance the E3 ligase function of MDM2. MDM4-null mice and a Knock-in of its RING domain also showed p53-dependent embryonic lethality, indicating that MDM4 and its RING domain are essential to negatively regulate p53 activity [62]. Similar to MDM2, spontaneous tumorigenesis was observed in mice overexpressing *MDM4* [63], and elevated expression of MDM4 or gene amplification has been described in a number of cancers. Although deregulation of MDM4 has not been investigated in MM, it is interesting to note that this gene is located in the long arm of chromosome 1q, whose gain is present in approximately 60% of patients [64].

Besides MDM2 and MDM4, a number of other ubiquitin ligases have been identified to regulate p53 in vitro like PIRH2, COP1, ARF-BP1, CHIP, TRIM24 or E4F1 [11,65]. Amplification of some of them, like PIRH2, COP1 and TRIM24 has been observed in some tumors using data from the TCGA that do not include samples of MM. The putative relevance of alterations of these factors, or others known to regulate the transcriptional activity of p53, such as WWP1, p300/CBP or ASPP [66] on p53 functionality in MM needs to be investigated.

## 4. Deregulation of p53 through Epigenetic Modifications

### 4.1. TP53 DNA Methylation

DNA methylation is a well-known epigenetic modification that decreases the expression of thousands of genes. The most investigated is methylation of the cytosines present in CpGs often grouped in CpG islands. Coding genes sequences as well as their promoters or 3′ untranslated regions (3′-UTRs) can be methylated, which abolishes gene transcription until a complex process of demethylation restores the expression [67]. Interestingly, DNA methylation pattern changes during tumorigenesis of many cancers including MM [68].

The first experimental evidence for p53 regulation by DNA methylation was provided by Schroeder et al. in 1997 [69], who observed that hypermethylation in the promoter region of *TP53* reduced the expression level of a reporter gene. These results were later confirmed by Pogribny et al. [70]. Since then, DNA methylation of *TP53* gene or its regulatory sequences have been reported in many cancers including Ewing’s sarcoma [71], glioblastoma [72], acute lymphoblastic leukemia (ALL) [73,74], human hepatocellular carcinoma [75], ovarian cancer [76], breast cancer [77] and also in MM [78,79]. On the contrary, lack of DNA methylation of *TP53* has also been found in other diseases like cutaneous squamous cell carcinomas [80], sporadic adrenocortical cancers [81] and myelodysplastic syndromes (MDS) [82]. Changes in DNA methylation of *TP53* may have important implications for tumor biology. In ALL, direct or indirect activation of the p53 pathway was observed after treatment with the demethylating reagent 5-aza-2′-deoxycitidine, and was accompanied by an increase in apoptosis [73] and in breast cancer, telomere shortening was correlated with a different level of hypermethylation of *TP53* [83]. On the contrary, in rectal cancer, methylation of p53 (investigated by methylation sensitive digestion) was correlated with apoptosis but not with p53 protein level [84]. Lack of correlation between p53 DNA methylation and protein level was also observed in glioblastoma multiforme [72].

Few studies in MM have addressed the putative influence of *TP53* methylation on protein expression. In some MM cell lines with p53 WT/− and no p53 protein expression, the second allele was found inactivated by promoter hypermethylation [78]. Additionally, treatment with demethylating agents increased the expression of p53 protein in several MM cell lines whose *TP53* promoter was hypermethylated [79]. More recently, it has been published that berberine, a compound with anticancer activity, induced apoptosis in the MM cell line U-266 through hypomethylation of the *TP53* promoter [85].

Mechanisms involved in regulation of *TP53* DNA methylation are complex and not well understood. In esophageal cancer cells, phospholipase C epsilon PLCE1 downregulation was linked to increased p53 level that was accompanied by reduced *TP53* promoter methylation [86]. In MM, the interleukin IL-6 seems to be one of the factors controlling p53 protein level and possibly *TP53* methylation [87].

Although DNA methylation is first and foremost linked to decreased gene expression, growing body of evidence suggest other effects of this epigenetic modification. Thus, the *TP53* mutation has been shown to correlate with *TP53* hypomethylation status of exons 5–8 [88,89]. Interestingly, methylation of *TP53* exons 5–8 can be increased in rat liver and colon mucosa by dietary selenomethionine, with may then influence the preservation of intact *TP53* sequence [90]. Whether similar effect can be achieved in humans and could be translated to chemoprevention of tumors remains unanswered.

### 4.2. miRNAs that Regulate the Activity of p53

MicroRNAs (miRNAs) are small non-coding RNAs that regulate gene expression at the post-transcriptional level. Over the past decade, the crucial role of miRNAs in the pathogenesis of many cancers has been clearly demonstrated. In particular, miRNAs are functionally deregulated in MM cells, and several studies reveal the involvement of miRNAs in MM pathophysiology. Moreover, the therapeutic potential of miRNA modulation has been investigated not only to target myeloma cells, but also to impair the functional interaction between myeloma cells and bone marrow microenvironment [91,92,93,94]. Recent studies have demonstrated that miRNAs interact with p53 and its network at multiple levels. Thus, p53 regulates the transcription, expression and the maturation of a group of miRNAs [95,96,97,98,99,100]. On the other hand, miRNAs can regulate p53, and in general gene expression, mainly by interacting with 3′-UTRs of the mRNAs leading to either mRNA degradation or inhibition of protein translation [95,101,102].

miR-504 was the first miRNA identified and validated by Hu et al. as a negative regulator of p53 [103]. This miRNA was showed to directly bind to *TP53* 3′-UTR site, which resulted in a decreased p53 expression in several types of cells. Similarly, other microRNAs such as miR-125b, miR-125a and miR-1285 have been described as negative regulator of *TP53* [104,105,106]. Interestingly, miR-612, which has the same seed sequence as miR-1285, cannot bind to the 3′-UTR of *TP53*, highlighting the tight regulation of p53 by miRNAs [106].

Up to now, only few miRNAs targeting *TP53* directly have been validated in MM cells. Two miRNAs, miR-25 and miR-30d, which directly interact with the 3′-UTR of the human *TP53* mRNA [107] are downregulated in MM and their levels are inversely correlated to *TP53* mRNA. When these miRNAs were reintroduced into MM cells, p53 targets such as *p21*, *BAX*, *PUMA* and *GADD45A* were activated, and reduced cell apoptosis, cell cycle arrest and senescence were observed. The miR-125a-5p which has been described to be upregulated in a subset of MM patients carrying the *t*(4;14) translocation [108], directly binds to *TP53* mRNA 3′-UTR triggering a decrease of p53 levels. On the other hand, its inhibition restored the p53 transcriptional pathway in p53 *WT* MM cells and also increased the level of miRNAs regulated by p53. Interestingly, inhibition of miR-125b was demonstrated to overcome dexamethasone resistance in MM cells by activation of p53 downstream targets [109]. It is important to note that several miRNAs deregulated in MM have been validated to directly target *TP53* 3′-UTR in different cellular models [110,111]. For example, miR-15a/miR-16-1 cluster in CLL [112], miR-19b in distinct types of cancer cell lines [113] or miR-375 in gastric cancer cells [114].

Several miRNAs have also been described to indirectly regulate p53 by affecting some of its regulators [96,115]. For example, miR-34a was shown to negatively regulate SIRT1, a protein that deacetylates p53 limiting its ability to transactivate its target genes [116]. As a consequence of miR-34a expression, p53 pathways are activated. This miRNA could be of particular interest in MM, since it was induced in MM cells by treatment with dexamethasone, which suppressed SIRT1 deacetylase and consequently allowed p53 activation [109]. Furthermore, miR-34a has been found to be a component of the *TP53* transcriptional network, with higher expression in WT *TP53* than in *TP53*-mutated MM cells, after treatment with nutlin-3, a known p-53 reactivating agent [117].

In MM, two studies have demonstrated indirect p53 regulation through miRNAs. Pichiorri et al. observed that the ectopic expression of miRNA-192, -194 and -215 induced significant down-regulation of MDM2 that was accompanied by p53 overexpression and p21 activation [97]. These miRNAs can, in turn, be transcriptionally activated by p53 demonstrating the auto regulatory loop between miRNAs and p53. In this regard, we have also demonstrated that miR-214 activates p53 by targeting PSMD10 that encodes the oncoprotein gankyrin, which negatively regulates p53 by enhancing its proteasomal degradation [118]. Specifically, using gain-of-function experiments and luciferase reporter assays we observed that ectopic transfection of miR-214 decreased the level of gankyrin protein by directly targeting PSMD10 3′-UTR. Moreover, as a consequence of gankyrin down-regulation in cells with WT *TP53*, an increase of *TP53* mRNA levels and subsequent up-regulation of CDKN1A (p21Waf1/Cip1) and BAX transcripts, which are direct transcriptional targets of p53, were observed. Taken together, the reported findings suggest an important role for miRNAs in the regulation of the p53 tumor suppression.

## 5. Altered Pattern of Human p53 Isoforms

Although, originally, p53 was considered a single protein, an alternative C-terminally-spliced isoform was soon described in mouse [119]. Several *TP53* isoforms were later discovered in humans. Decades of research on p53 isoforms have established that a p53-mediated cell response is the sum of the intrinsic activities of the coexpressed p53 isoforms and that alterations of their expression may also trigger p53 pathway deregulation, resulting in cancer and other diseases [120]. At least twelve p53 protein isoforms has been described encoded by nine p53 mRNAs (p53α, p53β, p53γ, Δ40p53α, Δ40p53β, Δ40p53γ, Δ133p53α, Δ133p53β, Δ133p53γ, Δ160p53α, Δ160p53β, and Δ160p53γ). The p53 protein isoforms are a consequence of alternative promoter usage (proximal and internal promoter), four alternative initiation codons (ATG1, ATG40, ATG133 and ATG160) and alternative splicing of introns 2 and/or 9, the last producing distinct C-terminal domains (α, β or γ) (Figure 1) [121,122,123]. All twelve p53 proteins isoforms share a common region of the DNA-binding domain but they have different transactivation and C-terminal domains [122], which allow them to differentially regulate the expression of different *TP53* target genes.

In the last years, numerous studies have demonstrated that p53 isoforms are differentially expressed in several human cancers types: breast cancer, colon carcinoma, melanoma, renal cell carcinoma (RCC), head and neck tumors (HNSSCs), hepatic cholangiocarcinoma, acute myeloid leukemia (AML), ovarian cancer, lung tumor, and glioblastoma [120,121,122,124,125,126]. Additionally, an association between the expression of p53 isoforms and clinical response and prognosis has been described [121]. These observations strongly suggest that deregulation of the expression of p53 isoforms may affect tumorigenesis. For example, in the malignant transformation from colorectal adenoma to carcinoma, a correlation exists between the increase of Δ133p53α and the decrease of p53β isoforms [127]. In AML, a clear interrelation of p53 isoforms profile with clinical outcome [128] has been demonstrated, and, in melanoma, high levels of p53β and Δ40p53α isoforms were expressed in tumor cells but not in melanocytes or fibroblasts [129].

Notably, various studies in different types of human cancers has revealed that *TP53* mutation status have superior prognostic value when is combined with the analysis of p53 isoform expression. Thus, the expression of p53 isoforms can modify the effects of *TP53* mutations in breast cancer and determination of p53γ isoform expression in *TP53* mutant breast cancer patients allows the identification of those with poor prognosis [124]. In serous ovarian cancer, patients with mutated *TP53* and Δ133p53α have a better overall survival than those with mutant *TP53* but no Δ133p53α isoform expression [130].

To date, there are no studies regarding the expression of p53 isoforms in MM, neither its putative clinical implication. Since mutation of *TP53* is not a frequent event at diagnosis, it would be interesting to explore this mechanism of p53 regulation in MM to evaluate its potential impact on response to treatment and disease prognosis.

## 6. p53-Based Antitumor Therapy

Despite, in the last years, the introduction of novel therapeutic agents with different mechanisms of action has improved considerably the median survival of patients, MM remains incurable [131,132,133]. Therefore, the investigation of other therapeutic agents is still needed.

Many different therapeutic strategies have been reported to reactivate or restore the p53 response in human cancers [25,134,135,136,137,138]. The first approach involves the modulation in different ways of the WT p53 activity, and the second have the general purpose to restore the functions of WT p53 in cells with *TP53* gene mutations [139].

Owing to the fact that p53 protein levels is under the tight control of two negative regulators, MDM2 and MDM4, several approaches to activate p53 have tried to inhibit the association of the protein with their negative regulators. This strategy is especially interesting in those tumors overexpressing *MDM2* or *MDM4*. The first molecule identified as a potent inhibitor of the p53-MDM2 interaction was nutlin [140]. This molecule displaces p53 from the binding on MDM2, causes the stabilization and accumulation of p53 protein, and subsequently leads to non-genotoxic activation of p53 pathway in cancer cells. Nutlins are effective in many types of cell lines but their efficacy requires WT status of the *TP53* gene and intact p53 signaling machinery [140,141,142]. Nutlin-3, the most used nutlin, has shown a potent antitumoral effect through the induction of apoptosis in different hematological tumor cells lines, such as ALL, AML, CLL, Hodgkin’s lymphoma and MM [78,139,143,144]. Interestingly, nutlin, the nutlin-derivative RG7112 and other compounds that antagonize MDM2 function such as MI-773, SAR405838, CGM097, MK-8242 and RO5503781, are all in phase I clinical trials [142,145,146,147]. Other small molecules that behave as MDM2 inhibitors and are being evaluated are benzodiazepines [148] and spiro-oxindole [149,150].

In MM, nutlin-3 has demonstrated potent antimyeloma activity in WT *TP53* cells in vitro and ex vivo, through the induction of p53 downstream targets like p21, MDM2, and the pro-apoptotic genes, *BAK*, *BAX* and *PUMA* [78,143,151]. Furthermore, synergy with various chemotherapeutic drugs commonly used in MM, namely, melphalan, etoposide and velcade has been found [143,152].

RITA (Reactivation of p53 and Induction of Tumor Cell Apoptosis) is other investigated compound identified in a cell-based screen. In contrast to nutlin, which binds to MDM2, RITA binds to the N-terminal domain of p53. This interaction causes conformational changes in the structure of p53 protein that reduce their affinity for MDM2 [137,153]. However, this first mechanism described for RITA is currently controversial in light of nuclear magnetic resonance (NMR) results that do not support this RITA-p53 interaction [154]. Anyhow, RITA has been described to bind to different proteins, triggering the DNA damage response [155,156] and inducing higher apoptosis than nutlin-3 [157]. In MM, RITA has been found to induce cell cycle arrest and apoptosis in cells that became resistant to other therapies, and even in cells with mutant or truncated *TP53*, suggesting the co-existence of an additional, p53-independent, mechanism of action [158,159]. A detailed review of approaches targeting p53-MDM2-MDM4 interactions was provided by Wade et al. [160].

The second p53-based therapeutic approach is focused on restoring the functions of p53 in cells with *TP53* gene mutations. PRIMA-1 (p53 reactivation and induction of massive apoptosis) is one the p53 mutant reactivating agents that have been developed. It reactivates mutant p53 by restoring its WT conformation and transcriptional functions, and enhances massive apoptosis in tumor cells [144]. Lambert et al. demonstrated that PRIMA-1 is converted to compounds that form adducts with thiols in mutant p53 and this modification of the mutant protein is sufficient to induce apoptosis in tumor cells [161]. PRIMA-1 ^MET^, the more potent derivative, can induce cell death independently of p53 and has been found to restore mutant p53 (R273H and R175H) function in vitro and in vivo [142,162,163]. This drug has been shown to have good efficacy against small cell lung carcinoma [164], breast cancer [165], thyroid cancer [166], acute promyelocytic leukemia [167], high-grade serous ovarian cancer [168], acute myeloid leukemia cells [169], epithelial ovarian cancer cells [170], soft-tissue sarcomas cell independent of p53 [171], and multiple myeloma [136,144,172,173]. However, most of these reports correspond to in vitro or short-term in vivo studies using xenograft models. Further investigations are then needed before this therapy can be transfer to clinical practice [174]. Interestingly, a p53-independent mechanism of PRIMA-1 in MM cell lines [172,173], has been reported suggesting that PRIMA-1 induces multiple signaling pathways to promote apoptosis. MIRA-1 is a maleimide compound, structurally distinct to PRIMA-1 but with higher potency [175]. Although it was initially identified as a mutant p53-reactivating small molecule, there are evidences that the cytotoxicity of this compound is mediated by a caspase-9-dependent apoptosis, while is independent of the *TP53* mutation status [176]. MIRA-1 has been reported to exhibit in vitro and in vivo anti-myeloma activity and synergism with dexamethasone, velcade and doxorubicin [177].

Finally, it is important to underscore that although p53 restoration remains an attractive approach for certain cancers, including MM, its ultimate therapeutic value can be affected by different pitfalls. Thus, the potential clinical use of nutlin could be hindered by the observed acquisition of somatic mutations in p53 and select for p53-mutated cells after nutlin treatment [178]. In this line, other studies point out that reactivation of p53 in established tumors may lead to variable and incomplete tumor regression, due to the acquire resistance to p53 functional restoration and the stage-heterogeneity of tumor cell populations [179,180]. In addition, it has been described that restoration of p53 in Mdm2-overexpressing angiosarcomas suppressed cell proliferation but did not induce apoptosis [181].

## 7. Conclusions

Inactivation of the p53 pathway occurs in the majority of human cancers and usually confers resistance to therapy and poor survival. Although mutations and deletions in the *TP53* locus are the most common alterations in p53 pathway, a number of other molecular mechanisms can also attenuate the p53 function. In fact, growing evidence of indirect p53 deregulation in MM through MDM2 overexpression, *TP53* promoter hypermethylation and alterations in certain miRNAs that directly or indirectly affect p53 expression, such as miR-25, miR-30d, miR-125a-5p and miR-214, have been reported. However, the impact of other known p53 negative regulators or variations in isoform expression in p53 inactivation remains to be elucidated. A systematic study of p53 status that integrates all these parameters would probably increase the proportion of MM patients exhibiting a dysfunctional p53 pathway. Therefore, the determination of p53 status in clinical studies would strongly contribute to a better management of cancer and could support the rationale for clinical trials with p53-based therapy.
